# Genome-wide identification and transcript analysis of TCP transcription factors in grapevine

**DOI:** 10.1186/s12864-019-6159-2

**Published:** 2019-10-29

**Authors:** Xiangpeng Leng, Hongru Wei, Xiaozhao Xu, Sandip A. Ghuge, Dongjie Jia, Gengsen Liu, Yongzhang Wang, Yongbing Yuan

**Affiliations:** 1 Qingdao Key Lab of Modern Agriculture Quality and Safety Engineering, College of Horticulture, Qingdao Agricultural University, Changcheng Road 700, Qingdao, 266109 People’s Republic of China; 2 Institute of Plant Sciences, The Volcani Center, Agricultural Research Organization, 50250 Bet-Dagan, Israel

**Keywords:** Grapevine, TCP transcription factors, Fruit development and ripening, Expression profiles analysis

## Abstract

**Background:**

The plant-specific TCP transcription factors play different functions in multiple processes of plant growth and development. TCP family genes have been identified in several plant species, but no comprehensive analysis of the TCP family in grapevine has been undertaken to date, especially their roles in fruit development.

**Results:**

A total of 18 non-redundant grapevine TCP (*VvTCP*) genes distributing on 11 chromosomes were identified. Phylogenetic and structural analysis showed that *VvTCP* genes were divided into two main classes - class I and class II. The Class II genes were further classified into two subclasses, the CIN subclass and the CYC/TB1 subclass. Segmental duplication was a predominant duplication event which caused the expansion of *VvTCP* genes. The cis-acting elements analysis and tissue-specific expression patterns of *VvTCP* genes demonstrated that these *VvTCP* genes might play important roles in plant growth and development. Expression patterns of *VvTCP* genes during fruit development and ripening were analyzed by RNA-Seq and qRT-PCR. Among them, 11 *VvTCP* genes were down-regulated during different fruit developmental stages, while only one *VvTCP* genes were up-regulated, suggesting that most *VvTC*P genes were probably related to early development in grapevine fruit. Futhermore, the expression of most *VvTCP* genes can be inhibited by drought and waterlogging stresses.

**Conclusions:**

Our study establishes the first genome-wide analysis of the grapevine *TCP* gene family and provides valuable information for understanding the classification and functions of the *TCP* genes in grapevine.

## Background

TCP proteins are a small family of plant-specific transcription factors and play important roles in multiple processes of plant growth and development by regulating cell growth and proliferation [1–3]. TCP transcription factors were named after four founding members: TEOSINTE BRANCHED1 (TB1) from *Zea mays*, CYCLOIDEA (CYC) from *Antirrhinum majus*, PROLIFERATING CELL NUCLEAR ANTIGEN FACTOR 1 and 2 (PCF1 and PCF2) from *Oryza sativa* [4–6]. TCP proteins are featured by the TCP domain, a highly conserved 59-residue-long basic helix-loop-helix (bHLH) structure at the N-terminus, which is associated with DNA binding, protein-protein interaction and protein nuclear localization [7]. Based on the sequence features and homology of the TCP domains, TCP family members were classified into two subfamilies: Class I (represented by the PCF proteins) and class II (represented by CYC and TB1) [2, 8]. The most noticeable difference between these two subfamilies is that class I members show a four-amino acids deletion in the basic region of the TCP domain [2]. The class II TCP members are further subdivided into two subclades (CIN and CYC/TB1) based on the difference among their TCP domain. Furthermore, several class II members have an arginine-rich motif (R domain) with unknown functions, which is hypothesized to be involved in facilitation of protein-protein interaction [1, 2].

Increasing evidences show that TCP transcription factors play versatile functions in multiple physiological and biological processes during plant growth and development, such as branching [9, 10], leaf morphogenesis [11, 12], flower development [13, 14], seed germination [15, 16], hormone pathways [17, 18] and response to environmental stress [19]. In *Arabidopsis*, *AtTCP14* and *AtTCP15* have been shown to regulate embryonic growth during seed germination by gibberellin signaling pathway [16]. They also could regulate leaf shape and internode length by promoting cell proliferation [12]. *AtTCP16* is observably expressed in developing microspores, and its down-regulation generated 50% abnormal pollen in transgenic plants [20]. Recently, strong experimental evidence supports that class I members of TCP proteins could be implicated in fruit development and ripening [21, 22]. Three tomato TCP genes (*SlTCP12*, *SlTCP15* and *SlTCP18*) are preferentially expressed in the tomato fruit and their expressions are regulated by ripening-related transcription factor, such as *RIPENING INHIBITOR* (*RIN)* and *COLORLESS NON-RIPENING* (*CNR)* [21]. The strawberry FaTCP11 gene participates in ripening-related processes and regulates flavan-3-ols synthesis [23].

The functions of most class II members of TCP family have been elucidated. For example, the *TB1* gene involves in the fate of maize axillary meristems [5] and the *CYC* gene affects the asymmetry, size and cell types of petals and stamens in *Antirrhinum* flower [4]. In *Arabidopsis*, *AtTCP18* and *AtTCP12*, two homologs of *TB1*, are involved in suppressing bud outgrowth [9]. The tomato orthologs *SlTCP9* (*SlBRC1a*) and *SlTCP7* (*SlBRC1b*) also show similar functions in axillary bud initiation and outgrowth [24]. *AtTCP1*, the homolog of *CYC*, mediates plant growth and development by regulating the expression levels of brassinosteroid biosynthesis gene DWARF4 [25]. Five *CIN*-like genes including *AtTCP2*, *AtTCP*3, *AtTCP4*, *AtTCP10* and *AtTCP24* were targeted by miR319 and have been shown to be involved in regulating leaf and flower development [14, 26–28]. Moreover, *AtTCP3* can increase flavonoid biosynthesis by interacting with R2R3-MYB proteins [29] and dominant-negative variant of *AtTCP3* leads to shorter and crinkled siliques [30]. Transient over-expression of *FvTCP9* in strawberry fruits dramatically promotes the expression of a series of genes involved in fruit color and aroma metabolism, suggesting that class II member of TCP family could be participated in fruit development and ripening processes [31].

To date, a number of *TCP* family members have been characterized in both dicots and monocots with the completion of entire genome, such as *Arabidopsis* [32], tomato [21], apple [33], strawberry [31], bamboo [34] and switchgrass [35]. However, little is known about the TCP family in grapevine [36], which is one of the most important fruit crop growing around the world with great nutritive and commercial value [37–39]. Due to the important roles of TCP transcription factors during plant growth and development, we performed for the comprehensive analysis of the *VvTCP* transcription factor family in grapevine. In the present study, 18 non-redundant *TCP* genes were identified from grapevine and were subsequently performed a systematic analysis including chromosome location, phylogenetic relationships, gene structure, conserved motif and *cis*-acting elements. We further analyzed the expression of *VvTCP* genes in diverse tissues, different stages of fruit development and ripening, as well as in response to hormones and stress treatment. This study provides reliable investigation of the *VvTCP* gene family and facilitates further functional characterization of *TCP* members in grapevine.

## Methods

### Identification of putative *VvTCP* in grapevine

Two different methods were peformed to identify and annotate TCP genes in grapevine genome. Firstly, the hidden Markov model (HMM) profile of the conserved TCP domain (PF03634) was downloaded from the Pfam database (http://pfam.janelia.org) and used to screen all grapevine proteins in the 12× coverage assembly of the *V. vinifera* PN40024 genome. Secondly, all *Arabidopsis* TCP protein sequences, which were downloaded from the *Arabidopsis* Information Resource (TAIR) database (http://www.arabidopsis.org), were used as queries to screen against grapevine genome database by using DNAtools software. Subsequently, all non-redundant VvTCP protein sequences were further verified for the presence of the TCP domain by screening against the Pfam (http://pfam.sanger.ac.uk/), InterProScan (http://www.ebi.ac.uk/Tools/pfa/iprscan/) and SMART (http://smart.embl-heidelberg.de/) database. The molecular weights (MW), isoelectric points (pI) and grand average of hydropathicity (GRAVY) of VvTCP proteins were calculated by the ExPasy website (https://web.expasy.org/protparam/). The subcellular location of VvTCP proteins was predicted by WoLF PSORT (http://www.genscript.com/psort/wolf_ psort.html).

### Sequence alignment and phylogenetic analysis

Sequences of the 24 *Arabidopsis* and 22 rice TCP proteins were retrieved from TAIR (https://www.arabidopsis.org/) and rice genome database (http://rice.plantbiology.msu.edu/), respectively. The sequences of 30 tomato TCP family members were retrieved from the Solanaceae Genomics Network (https://solgenomics.net/). The sequences of 19 strawberry TCP family members were retrieved from PlantTFDB (http://planttfdb.cbi.pku.edu.cn/). The *Antirrhinum* CYC and maize TB1 were retrieved from NCBI database (https://www.ncbi.nlm.nih.gov/).

ClustalX 2.0 software was used to perform the multiple sequence alignments of the amino acid sequences of the TCP proteins of grapevine, *Arabidopsis*, rice, tomato and strawberry. An unrooted phylogenetic tree based on the full length protein sequences sequence alignments was constructed using MEGA 7.0 software and the neighbor-joining method with the following parameters: pairwise alignment, 1000 bootstrap replicates, Poisson correction model, uniform substitution rates and complete deletion. Moreover, another phylogenetic tree was also constructed using all protein sequences of TCP domain in grapevine for further analysis. The motif logos of the VvTCPs were generated by submitting the sequences to the MEME website (http://meme.nbcr.net/meme/cgi-bin/meme.cgi). Below are the parameters of MEME used: maximum number of motifs, 20; minimum motif width, 6; and maximum motif width, 50.

### Chromosomal location, gene structure, and duplication analysis

All *VvTCP* genes were mapped to grapevine chromosomes based on physical positions at the Grape Genome CRIBI website (http://genomes.cribi.unipd.it/) and the map was drawn using the MapInspect software. Accordingly, the cDNA sequences and their corresponding genomic DNA sequences of *VvTC*P members were obtained from the grapevine genome, then the exon-intron organization were identified by comparing the coding sequences with their corresponding genomic sequences using the GSDS software (http://gsds.cbi.pku.edu.cn) [40]. Tandem duplicated genes were defined by checking their physical locations on individual chromosomes and were identified as adjacent paralogous on a grape chromosome, with no more than one intervening gene [41]. For synteny analysis, the synteny blocks were detected by MCScanX software (http://chibba.pgml.uga.edu/mcscan2/), with the E-value set below 1 × 10^− 5^ taking reference from a previous study [42]. The diagrams were generated by the program Circos version 0.63 (http://circos.ca/) [43].

### In silico promoter analysis

The promoter sequences of 1, 500 bp upstream of the coding region of each *VvTCP* genes were retrieved from the grapevine genome website CRIBI (http://genomes.cribi.unipd.it/). PlantCARE online program (http://bioinformatics.psb.ugent.be/webtools/plantcare/html/) were employed to search the putative *cis*-acting element [44].

### Expression profiles of *VvTCPs* in various organs and different berry developmental stages

The expression profiles of *VvTCP* genes were determined in a *Vitis vinifera* cv ‘Corvina’ (clone48) gene expression atlas of various organs at different developmental stages. Microarray data were obtained from the NCBI gene expression omnibus (GEO) datasets under the series entry GSE36128 (http://www.ncbi.nlm.nih.gov/geo/) [45]. The mean of expression value of each gene in all tissues/organs were analyzed and graphically represented using Multi Experiment Viewer (MeV) software [46]. The expression patterns of *VvTCP* genes in fruit developmental stages were acquired from gene expression omnibus (GEO) database of NCBI (GSE77218), which measured using RNA-sequencing (RNA-Seq) data [47]. Berries from 3 year old grapevine trees ‘Fujiminori’ (*V. vinifera*× *V. labrusca*) were sampled in triplicate at the green fruit expanding (40DAF or DAF40), veraison (65DAF or DAF65), and ripe (90DAF or DAF90) stages throughout the growing season. Furthermore, expression analyses of *VvTCP* genes in 10 different grapevine (*Vitis vinifera*) varieties at four berry development stages were based on RNA-seq data (accession numbers GSE62744 and GSE62745) downloaded from the NCBI GEO datasets [48]. The 10 varieties contained five red-skinned (Sangiovese, Barbera, Negro amaro, Refosco and Primitivo) and five white-skinned berries (Vermentino, Garganega, Glera, Moscato bianco and Passerina). Berries were sampled in triplicate at four developmental stages, the pea-sized berry stage at 20d after flowering, the berries beginning to touch stage just prior to veraison (Pre_veraison), the berry-softening stage at the end of veraison (End_veraison), and the fully ripe berry stage at harvest.

### The expression of *VvTCP* under stress condition

To investigate the expression profiles of *TCPs* in response to different stress treatment (Cu, salt, waterlogging and drought stress), grapevine RNA-seq data sets (SRA accession no. SRP070475 and SRP074162) were retrieved from NCBI GEO database (https://www.ncbi.nlm.nih.gov/geo/) or from published supplemental data sets [37, 49–51]. Two-year-old ‘Summer Black’ (hybrids of *V. vinifera* and *V. labrusca*) grapevine were used to investigate the expression of *TCP* genes in response to abiotic stresses. Cu stress of potted grapevine plants was simulated with 100 μM CuSO_4_ and salt stress was treated with 0.8% NaCl [37, 50]. The control plantlets were similarly treated with distilled water. Waterlogging treatment were performed by immersing the plants to water for 48 h [51] and drought treatment was performed by withholding water 20 days [49]. Grapevine plantlets grown in the standard conditions were used as a control. All types of samples were three replicates and the third and fourth unfolded leaves from the shoot apex was collected from treatment and control groups during deep sequencing. The analysis of RNA-seq data was according to previous method [37] and the RPKM (Reads Per Kilobase per Million mapped reads) values were used to estimate the gene expression level. The heatmap of *TCP* genes was exhibited using R software (http://www.bioconductor.org/).

### Plant growth condition and gene expression analysis using qRT-PCR

Four-years-old ‘Fujiminori’ grapevine trees, grown in the standard field conditions at the Qingdao Agricultural University fruit farm, Qingdao, China, were chosen as the experimental material. To investigate gene expression profiles of *TCP* genes during berry development and ripening, grapevine berry samples were also collected at three time points: the green fruit expanding stage (40 DAF), veraison (70 DAF) and ripe/harvest stages (90 DAF) throughout the growing season. All samples were collected in triplicate from each of the sampling points. The samples were immediately frozen in liquid nitrogen and stored at − 80 °C until use.

A total of 200 mg of the grapevine tissues were used from above mentioned samples for total RNA isolation using the modified CTAB method [38], followed by DNaseI (Tiangen, Beijing, China) digestion to eliminate any contaminating DNA. For qRT-PCR analysis, the first-strand cDNAs was synthesized from the 1 μg RNA using a PrimeScriptTM RT Reagent Kit (TaKaRa, Dalian, China) according to the manufacturer’s instructions. Expression pattern of various genes obtained from Microarray data was validated by qRT-PCR. The primers used for the qRT-PCR were designed using Primer 3.0 online and details of the primer sequences were presented in Additional file 2: Table S1. The grapevine housekeeping gene Actin (AB073011) was used as the internal control. The qRT-PCR was peformed using SYBR® Premixm Ex Taq™ (TaKaRa, Japan) with the Applied Biosystems 7500 Real-Time PCR System. All the experiments were carried out with three biological replicates. The 2^–ΔΔCT^ method was used to estimate the relative expression level [52].

### Subcellular localization of grapevine TCP genes

Based on the grapevine genome and public NCBI database, the full coding sequences of three randomly selected *VvTCP* genes were PCR-amplified with high-fidelity HS polymerase (TaKaRa Biotechnology, Dalian, China) using the primers listed in Additional file 2: Table S1. To construct green fluorescent protein (GFP)-tagged VvTCP, the three cloned *VvTCP* genes (35S, VvTCP2-GFP, 35S: VvTCP3-GFP and 35S: VvTCP18-GFP) were inserted into the pCAMBIA1300 vector, respectively. After electroporation of these construction into *Agrobacterium tumefaciens* EHA105, the transformed bacterial cells were activated and infected into the leaf tissue of *Nicotiana benthamiana* as previously described [53]. The transient expression of VvTCPs-GFP was observed 72 h later using a laser confocal microscope (Zeiss LSM700, Germany), the mCherry-labelled nuclear marker (NF-YA4-mCherry) was used to visualize the nucleus.

## Results

### Identification of *TCP* gene family in grapevine

In order to identify and obtain the TCP genes in grapevine genome, the BLAST searches were performed at NCBI and other public databases. Subsequently, the HMM profile was employed to perform a global search of the grapevine genome (http://genomes.cribi.unipd.it/grape/). After removing the redundant sequences, 18 non-redundant *VvTCP* genes were identified and mapped onto 11 out of 19 grapevine chromosomes (Additional file 1: Figure S1). Further, 18 *VvTCP* genes were annotated as *VvTCP1* to *VvTCP18* on the basis of their distributions in genome and relative linear orders among the respective chromosome.

Protparam tool was used to analyze the physical and chemical characterizations of the VvTCP proteins (Table 1). The length of VvTCP proteins varied from 169 to (VvTCP14) 460 amino acid residues (VvTCP9). VvTCP14 showed the lowest value of the molecular weight (17.72 kDa), while the highest of the molecular weight (48.54 kDa) was observed in VvTCP6. The values of theoretical isoelectric point (pI) ranged from 6.09 to 9.71. The value of the aliphatic index ranged from 56.37 to 80.36, which suggested that the VvTCP proteins contained rich aliphatic amino acids. The GRAVY of all VvTCP proteins was less than zero, indicating that VvTCPs were hydrophilic. The majority of VvTCP proteins were predicted to be located on the nucleus by WoLF PSORT, but a few of them may be located in other subcellular compartments, such as chloroplast and cytoplasm (Table 1).

**Table 1:** TCP gene family in grapevine

Gene Name	Accession number	Protein	Chrom	Chr srart	Chr end	MW(Da)	pI	Aliphatic index	GRAVY	Loc
VvTCP1	VIT_01s0011g0292.t01	438	Chr1	2,574,244	2,575,738	48,349.63	9.43	69.04	−0.562	nucl: 7.5, golg: 5, cyto_nucl: 4.5
VvTCP2	VIT_01s0026g0220.t01	353	Chr1	11,610,314	11,611,375	38,054.39	8.93	62.49	−0.627	nucl: 13
VvTCP3	VIT_02s0025g0459.t01	411	Chr2	4,140,127	4,141,512	43,499.20	6.20	69.59	−0.296	nucl: 13
VvTCP4	VIT_08s0040g0160.t01	204	Chr8	12,723,686	12,724,300	21,699.26	8.46	60.83	−0.507	nucl: 6, mito: 6, cyto: 2
VvTCP5	VIT_10s0003g0087.t01	382	Chr10	2,112,286	2,113,434	42,398.40	6.40	76.65	−0.519	nucl: 10, chlo: 1, cyto: 1
VvTCP6	VIT_10s0003g0391.t01	444	Chr10	6,666,048	6,667,382	48,535.28	7.84	58.02	−0.873	nucl: 13
VvTCP7	VIT_10s0042g0017.t01	255	Chr10	12,942,744	12,943,511	26,159.42	9.71	73.29	−0.234	nucl: 7, chlo: 3, mito: 3
VvTCP8	VIT_12s0028g0252.t01	307	Chr12	3,281,712	3,282,899	33,699.77	6.41	74.04	−0.386	nucl: 11, chlo: 2
VvTCP9	VIT_12s0035g0069.t01	460	Chr12	20,150,532	20,151,914	48,077.84	6.57	56.37	−0.668	nucl: 14
VvTCP10	VIT_14s0083g0015.t01	388	Chr14	22,124,744	22,125,983	44,040.07	9.57	68.43	−0.672	nucl: 10.5, cyto_nucl: 6.5, chlo: 2
VvTCP11	VIT_14s0068g0033.t01	349	Chr14	24,046,932	24,047,981	38,623.45	8.79	76.50	−0.573	nucl: 10.5, nucl_plas: 6, chlo: 1
VvTCP12	VIT_14s0068g0169.t01	296	Chr14	25,396,768	25,397,658	31,511.13	9.01	68.95	−0.625	nucl: 12, chlo: 1
VvTCP13	VIT_15s0048g0115.t01	339	Chr15	15,268,480	15,269,562	36,052.01	8.96	72.92	−0.343	nucl: 11, cyto: 2
VvTCP14	VIT_16s0022g0248.t01	169	Chr16	15,211,547	15,212,056	17,721.95	6.62	80.36	−0.307	nucl: 10, cyto: 3
VvTCP15	VIT_17s0000g0418.t01	366	Chr17	4,344,260	4,345,620	41,570.75	8.88	66.69	−0.757	nucl: 8, cyto: 3, chlo: 1
VvTCP16	VIT_17s0000g0602.t01	369	Chr17	6,588,791	6,589,900	39,568.76	7.20	58.73	−0.640	nucl: 14
VvTCP17	VIT_18s0117g0030.t01	355	Chr18	23,608,849	23,609,916	37,106.80	6.09	60.82	−0.555	nucl: 14
VvTCP18	VIT_19s0014g0168.t01	398	Chr19	1,805,797	1,806,993	43,306.70	6.27	58.19	−0.695	nucl: 14

*AA* amino acid residues, *Chrom* chromosome, *MW* molecular weight, *pI* theoretical isoelectric point, *GRAVY* grand average of hydropathicity, *Loc* subcellular location. The subcellular location results of grapevine BBX genes were predicted by WoLF PSORT (https://www.genscript.com/wolf-psort.html). *Nucl* nucleus, *Chlo* chloroplast, *Cyto* cytosol, *Mito* mitochondria. Testk used for kNN is: 14

### Phylogenetic analysis and classification of the VvTCP family

**Fig. 1:**
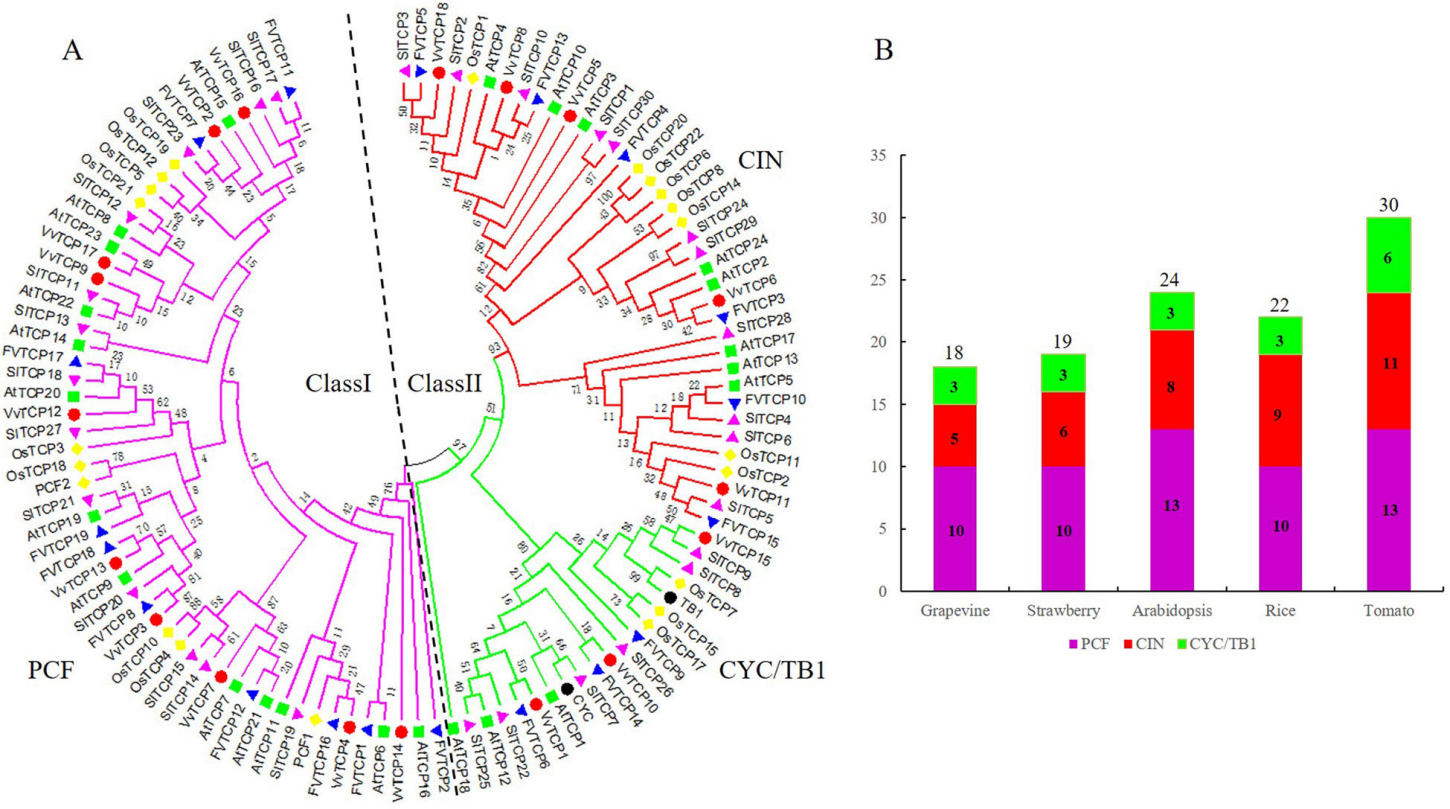
Phylogenetic analysis of TCP family among grapevine, strawberry, *Arabidopsis*, rice and tomato. **a** The full-length amino acid sequences of TCP from grapevine (VvTCP), strawberry (FvTCP), *Arabidopsis* (AtTCP), rice (OsTCP), tomato (SlTCP), the *Antirrhinum* CYC and maize TB1 were aligned by ClustalX, and the phylogenetic tree was constructed using the neighbor-joining method with 1000 bootstrap replicates by MEGA7.0. The branched lines of the subtrees are colored to indicate different TCP subgroups. **b** TCP family members of grapevine, strawberry, *Arabidopsis*, rice and tomato

**Fig. 2:**
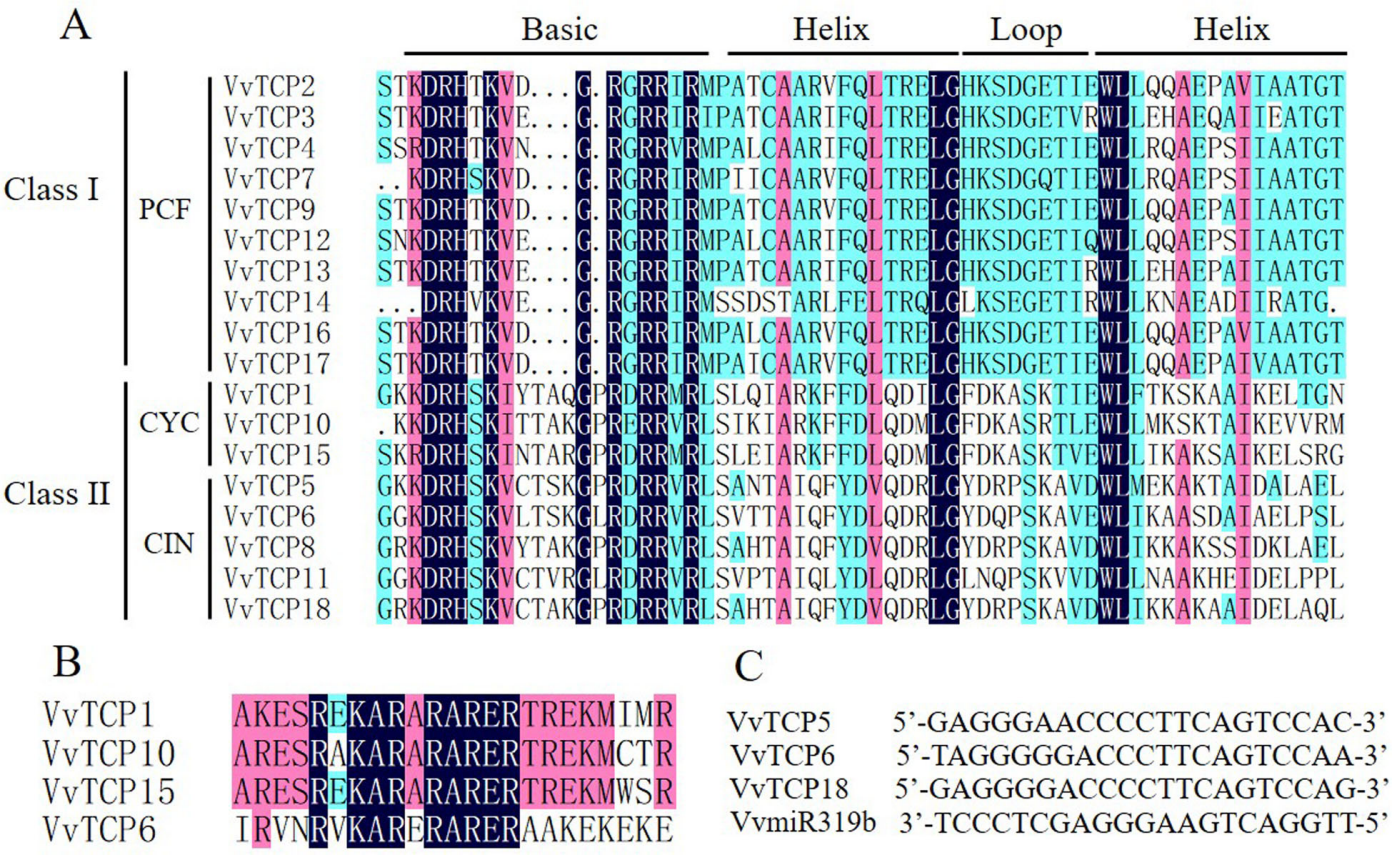
Multiple sequence alignment of grapevine TCP proteins. **a** Alignment of the TCP domain for the predicted grapevine TCP proteins. Overall conserved amino acids are in blue. **b** Alignment of the R-domain of class II subfamily members. **c** Alignment of putative target areas for miR319b (aligned in reverse)

To explore the evolutionary and phylogenetic relationships between grapevine TCP proteins and other known TCPs, the full length of 115 TCP proteins from grapevine, *Arabidopsis*, rice, strawberry, tomato and two TCP genes (*TB1* and *CYC*) with known function were used to construct a phylogenetic tree using Neiboring-Joining method (Fig. 1). Furthermore, in order to assess a better understanding of phylogenetic relationships of VvTCP members, multiple-alignment of the core TCP domain of the all VvTCPs was also performed. Both the phylogenetic analysis and TCP domain alignment suggested that the grapevine TCP proteins were classified into two classes: class I (or PCF) contained 10 genes and class II contained 8 genes (Figs. 1a and 2a). Four-amino-acid fewer in the basic domain of class I than class II proteins was the most striking difference observed between these two classes (Fig. 2a). Additionally, the phylogenetic tree showed that class II could be further divided into two subclades, CYC/TB1 and CIN (Figs. 1a and 2a). Furthermore, all *Arabidopsis*, rice, strawberry and tomato TCPs existed the same class or clade as previous reports [21, 31, 54], confirming the reliability of our phylogenetic tree. According to the classification, the CYC/TB1 subclade contained 3 *VvTCP* genes (*VvTCP1*, *VvTCP10* and *VvTCP15*) and the CIN subclade included 5 *VvTCP* genes (*VvTCP5*, *VvTCP6*, *VvTCP8*, *VvTCP11* and *VvTCP18*).

Expect for the TCP domain, several class II TCP members also share an R domain, which is an approximately 18-residues arginine-rich motif. As shown in Fig. 2b, four class II proteins, VvTCP1, VvTCP10 and VvTCP15 from grapevine class II CYC/TB1 as well as VvTCP6 from CIN, contained the R domain at the C-terminus of the TCP domain. The VvTCP6 in the CIN subclade was less conserved than CYC/TB1 subclade, in agreement with the previous in tomato and *Phalaenopsis equestris* [21, 55]. Additionally, three CIN subclade genes (*VvTCP5*, *VvTCP6* and *VvTCP18*) included the potential miR319 target site and displayed high sequence homology with the *Arabidopsis* and tomato miR319-targeted TCP genes (Figs. 1a and 2c).

### Gene structure analysis and conserved motif identification

**Fig. 3:**
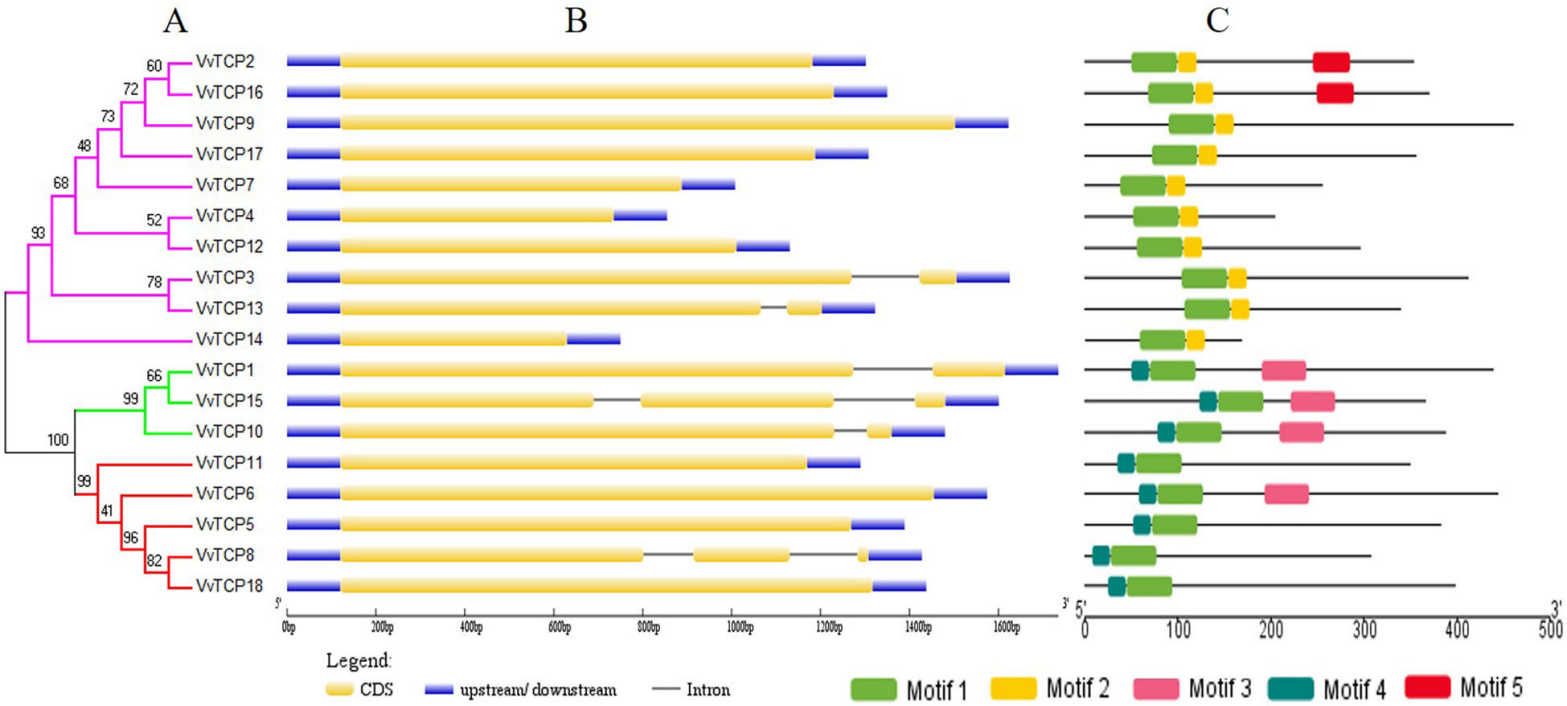
Phylogenetic analysis, gene structure and conserved motifs of TCP family in grapevine. **a.** The conserved TCP domain sequences of VvTCP proteins was constructed a Neighbor-Joining phylogenetic tree and the bootstrap test was performed with 1000 iterations. **b** Exon-intron structure of VvTCP genes. Blue indicates untranslated 5′- and 3′-regions, yellow indicates exons; black indicates intro ns. **c** Distribution of conserved motifs of VvTCP proteins. Different motifs are shown by different colors numbered 1 to 5. See legend for detailed color.

To further understand into the evolutionary relationships and structural features of the TCP protein in grapevine, the exon/intron structures and conserved motifs of VvTCPs were investigated. The conserved TCP domain sequences of VvTCP protein were used to construct a new phylogenetic tree, which also divided the VvTCP proteins into three subgroups (Fig. 3a). As shown in Fig. 3b, almost all *VvTCP* genes exhibited highly conserved exon-intron organization: 12 out of 18 *VvTCP* genes were no intron, four *VvTCP* genes had one intron, and two *VvTCP* genes had two introns. As expected, most of *VvTCP* genes within same subfamily exhibited similar distribution patterns of exon/intron in terms of exon length and intron number, which supported the classification of subclade and evolutionary relationship (Fig. 3b).

To get more insight into the diversity of motif compositions among VvTCPs, five conserved motifs were identified by MEME program. The results showed that the highly conserved TCP domain (motif 1) was existed in all VvTCP proteins (Fig. 3c and Additional file 1: Figure S2). The conserved R domain (motif 3) was hit in four class II VvTCP proteins. All class I members were characterized by motif 2 in C-terminal TCP domain. By comparison, the N-terminal TCP domain of motif 4 was detected in all class II proteins. Additionally, motif 5 were exclusively present in PCF, which was consistent with the previous report that some motifs existing in a particular subgroup may contribute to the specific function of those genes in the subgroup [31, 56]. Togerher, VvTCP proteins clustered in same subgroup demonstrated similar motif composition, which was in agreement with the gene structure analysis.

### Tandem duplication and synteny analysis of *VvTCP* genes

**Fig. 4:**
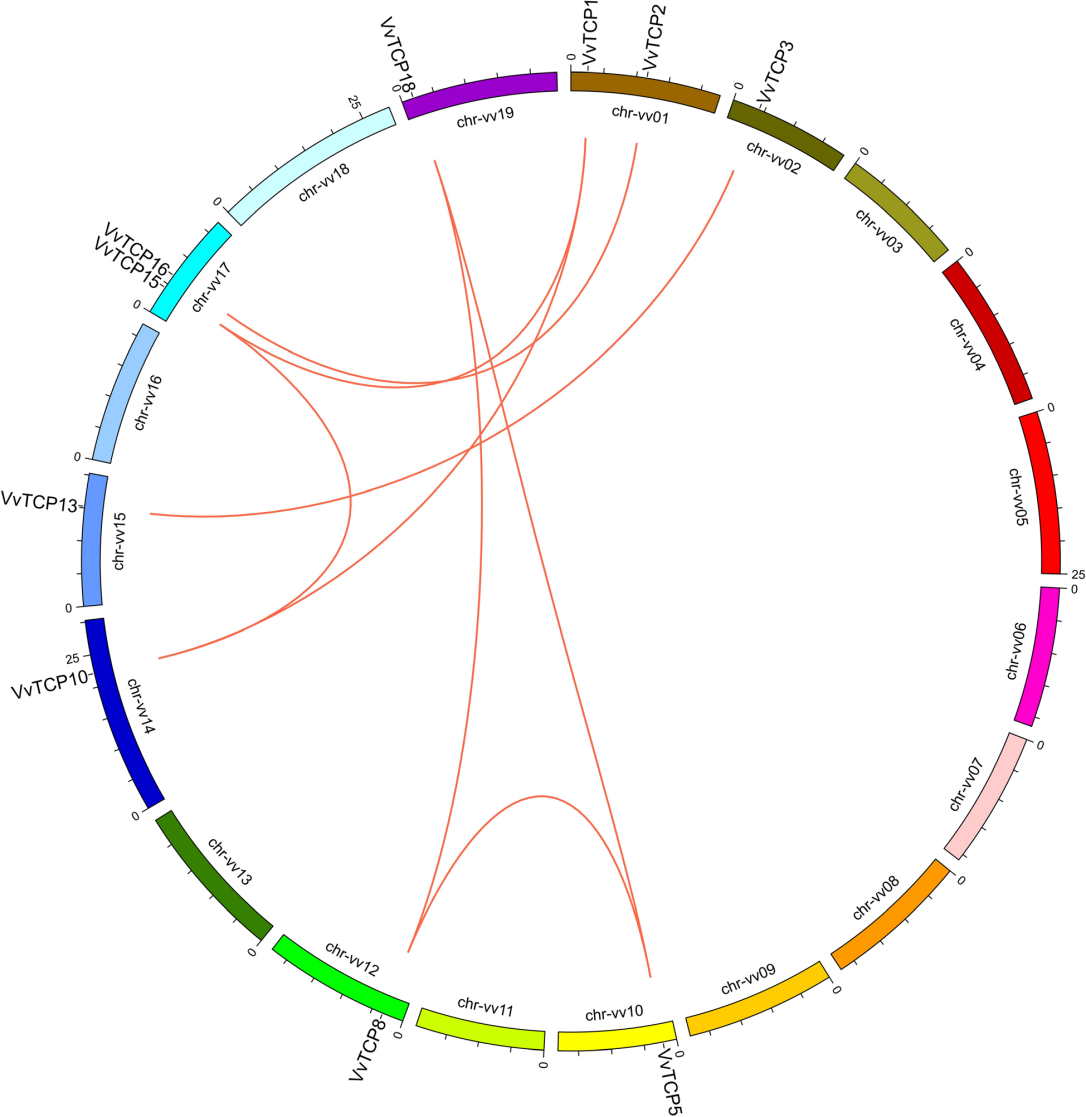
Chromosome distribution and synteny analysis of grape TCP genes. Chromosomes 1–19 are shown with different colours and in a circular form. The approximate distribution of each VvTCP gene is marked with a short line on the circle. Red curves denote the details of syntenic regions between grape TCP genes

To reveal the mechanism for expansion and evolution of the *VvTCP* gene family, potential gene duplication events were investigated in the of grapevine genome. As illustrated in Fig. 4 and Additional file 3: Table S2, eight pairs of paralogous *VvTCP* genes were identified and distributed on different chromosomes in grapevine, whereas no tandem duplication events were observed, suggesting that segmental duplications were the main causes for the amplification of *VvTCP* gene family. In addition, six genes involved in two segmental duplication events (*VvTCP1*/*VvTCP10*/*VvTCP15* and *VvTCP5*/*VvTCP8/VvTCP18*).

Furthermore, a large-scale comparative synteny maps between grapevine and *Arabidopsis*, grapevine and tomato was analyzed at genome-wide levels with purpose to clarify the origin and function of *TCP* genes. A total of eight pairs of *TCP* genes were identified between grapevine and *Arabidopsis* (Additional file 1: Figure S3 and Additional file 3: Table S2), while 37 pairs of *TCP* genes, including 12 *VvTCP* genes and 17 *SlTCP* genes, showed syntenic relationship (Additional file 1: Figure S4 and Additional file 1: Table S2), suggesting that most TCPs had orthologous in *Arabidopsis* and tomato. Among the synteny events between grapevine and tomato, 8 *VvTCP* genes were found to be associated with at least three synteny events, such as *VvTCP5*- *SlTCP1*/*SlTCP2*/*SlTCP3*/*SlTCP10*/*SlTCP30* and *VvTCP15*- *SlTCP7*/*SlTCP8*/*SlTCP9*/*SlTCP22*/*SlTCP25* (Additional file 3: Table S2). Interestingly, six out of these eight genes were in CIN and CYC/TB1 subclade, indicating a higher conservation of CIN and CYC/TB1 than PIF subclade in *TCP* gene family.

### Promoter *Cis*-regulatory elements analysis of grapevine *VvTCP* genes

**Fig. 5:**
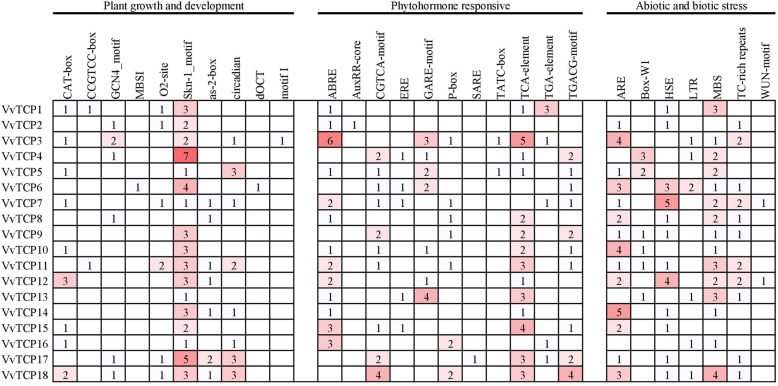
Promoter *Cis*-regulatory elements analysis of grapevine VvTCP genes. Number of each cis-acting element in the promoter region (1.5 kb upstream of the translation start site) of *VvTCP* genes. Based on the functional annotation, the cis-acting elements were classified into three major classes: plant growth and development, phytohormone responsive, or abiotic and biotic stresses-related cis-acting elements (detailed results shown in Supplementary Table S2)

To further insight into the gene function and regulation mechanism of *VvTC*P genes, the cis-regulatory elements in promoter sequences were analyzed. The promoter regions (1, 500 bp of genomic DNA sequence upstream of the translation starts site) of the VvTCP genes were submitted in PlantCARE database. In addition to the basic TATA and CAAT boxes, a large number of *cis*-acting elements involved in phytohormone responses, plant growth and development and stress responses were identified (Fig. 5; Additional file 4: Table S3). As show in Fig. 5, two *cis*-acting regulatory elements involved in endosperm expression (GCN4_motif and Skn-1_motif) were identified in promoter region of 6 and 17 *VvTCP* genes, respectively. Three *cis*-acting regulatory elements were related to meristem expression (CAT-box, CCGTCC-box and dOCT) in plant growth and development. The shoot-specific expression element (as-2-box) and circadian control element (circadian) were found in 7 and 8 *VvTCP* genes, respectively. Additionally, the flavonoid biosynthetic (MBSI), zein metabolism regulation element (O2 site) and root specific (motif I) regulatory element were also found in the promoter region of the *VvTCP* genes (Fig. 5; Additional file 4: Table S3).

In hormone-related *cis*-acting elements, the ABA-responsive element (ABRE), the salicylic acid (SARE and TCA-element), the MeJA-responsive element (CGTCA-motif and TGACG-motif) and the gibberellin-responsive element (P-box, GARE-motif and TATC-box) were identified in the promoter region of 13, 14, 10 and 13 *VvTCP* genes, respectively (Fig. 5; Additional file 4: Table S3). Ethylene-responsive element (ERE) and auxin-responsive element (AuxRR-core and TGA-element were observed in 5 and 6 *VvTCP* genes respectively (Fig. 5; Additional file 4: Table S3). Plenty of hormone-responsive elements were observed in the promoter region of *VvTCP* genes, revealing that hormones could play important functions in the regulation of plant growth and development (Fig. 5). In stress-related *cis*-acting elements, anaerobic induction (ARE), drought-inducibility (MBS), heat stress (HSE) and low-temperature (LTR) responsiveness element were also detected in the promoters of 14, 15, 12 and 6 *VvTCP* genes, respectively (Fig. 5; Additional file 4: Table S3).

### Tissue-specific expression patterns of *VvTCP* genes in grapevine

**Fig. 6:**
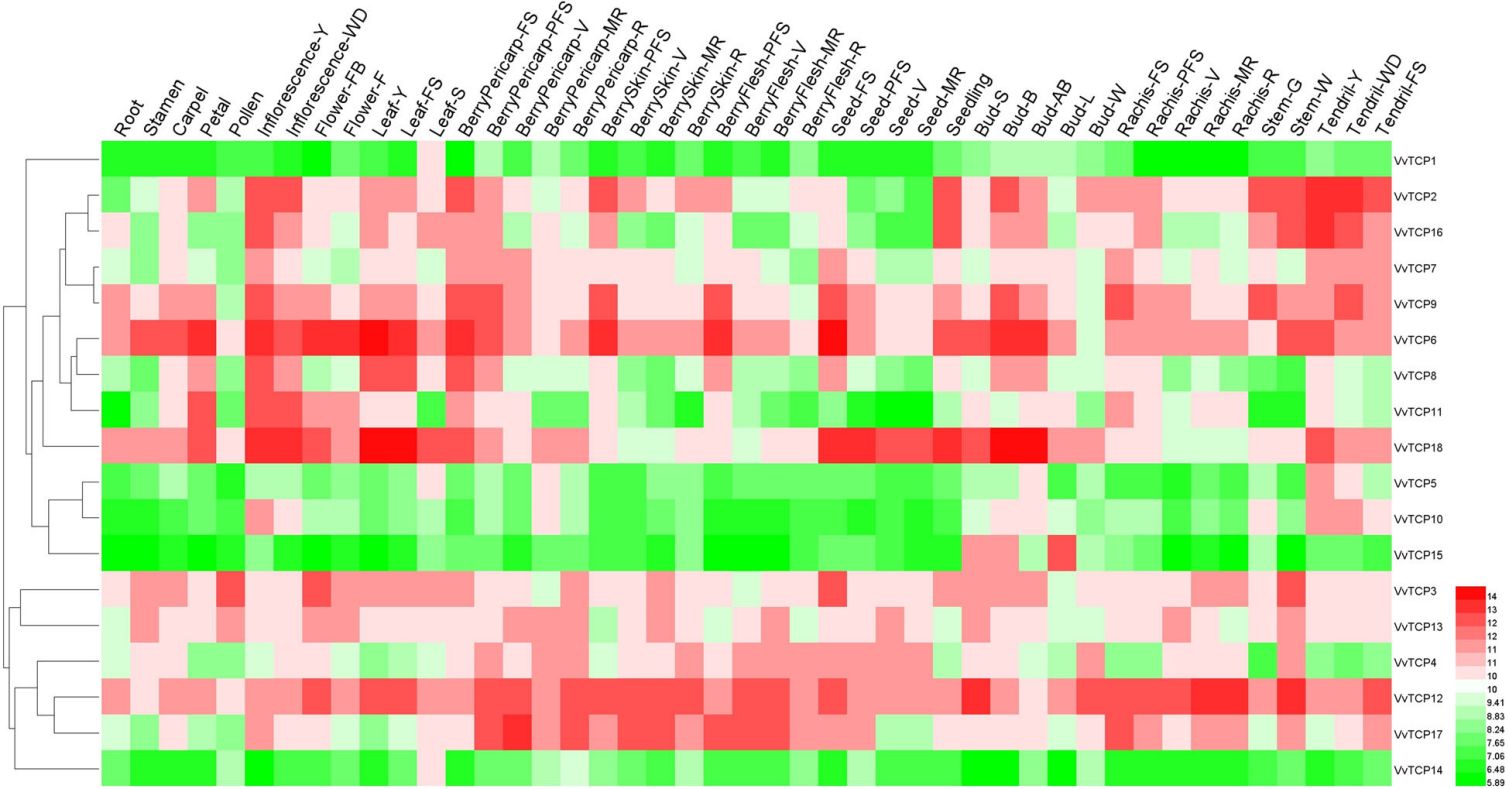
Expression profiles of grapevine *VvTCP* genes in various tissues and developmental stages. Expression data were normalized based on the mean expression value of each gene in all tissues analysed. Genes were hierarchically clustered based on average Pearson’s distance metric and ‘average linkage’ method. Red and green boxes indicate high and low expression levels, respectively, for each gene. Bud-AB, bud after burst; Bud-B, Bud burst; Bud-W, winter bud; Bud-L, latent bud; Bud-S, bud swell; Flower-F, flowering; Flower-FB, flowering begins; FS, fruit set; Inflorescence-Y, young inflorescence with single flowers separated; Inflorescence-WD, well-developed inflorescence; Leaf-FS, mature leaf; Leaf-S, senescing leaf; Leaf-Y, young leaf; MR, mid-ripening; R, ripening; PFS, post fruit set; Stem-G, green stem; Stem-W, woody stem; V, véraison

To gain more insights in potential roles of *VvTCP* genes during grapevine development, the organic-specific expression patterns of all the *VvTCP* genes were analysed using an expression atlas of *V. vinifera* cv. ‘Corvina’ from the GEO DataSets (GSE36128), which contained 42 various organs/tissues at different developmental stages obtained by microarray analysis [45]. Hierarchical clustering was used to present the relative expression levels of *VvTCP* genes in different tissues. As showed in Fig. 6, some *VvTCP* genes shared similar expression profiles in various tissues, while other *VvTCP* genes presented significant tissue-specific expression patterns, possibley suggesting the functional divergence of *VvTCP* genes in grapevine organs/tissues during development. For example, *VvTCP6* and *VvTCP12* were constitutively high expressed in almost all tested issue, whereas *VvTCP1*, *VvTCP5, VvTCP10* and *VvTCP14* were expressed at extreme low levels in all tissues (Fig. 6, Additional file 5: Table S4).

In contrast, the expression levels of *VvTCP2* and *VvTCP16* were very high in young inflorescence, seedling and woody stem and they were relatively low expression in seed and pollen, implying that they might be involved in the development of inflorescence, seedling and woody stem (Fig. 6, Additional file 5: Table S4). *VvTCP15* was only high relative expression level in latent bud, bud swell or bud burst and at almost undetectable levels in other tissues, suggesting that *VvTCP15* might plant an important role in the development of grapevine buds (Fig. 6, Additional file 5: Table S4). *VvTCP11* displayed high expression in petal, young inflorescence or well-developed inflorescence, indicating an involvement in flower development. Additionally, *VvTCP8* showed relatively high expression in young leaves and inflorescence, *VvTCP18* was extremely high transcript levels in young leaves, mature leaves, burst bud and bud after burst. Remarkably, some *VvTCP* genes (*VvTCP2*, *3*, *6*, *8*, *9* and *11*) were gradually decreased expression patterns from the green fruit stage to the veraison/ripe stage (Fig. 6, Additional file 5: Table S4), which indicated that these genes might play important roles in fruit development. These results prompted us to investigate the transcript accumulation patterns of *VvTCP* genes during grapevine fruit development and ripening.

### Expression patterns of *VvTCP* genes during different berry developmental stages

**Fig. 7:**
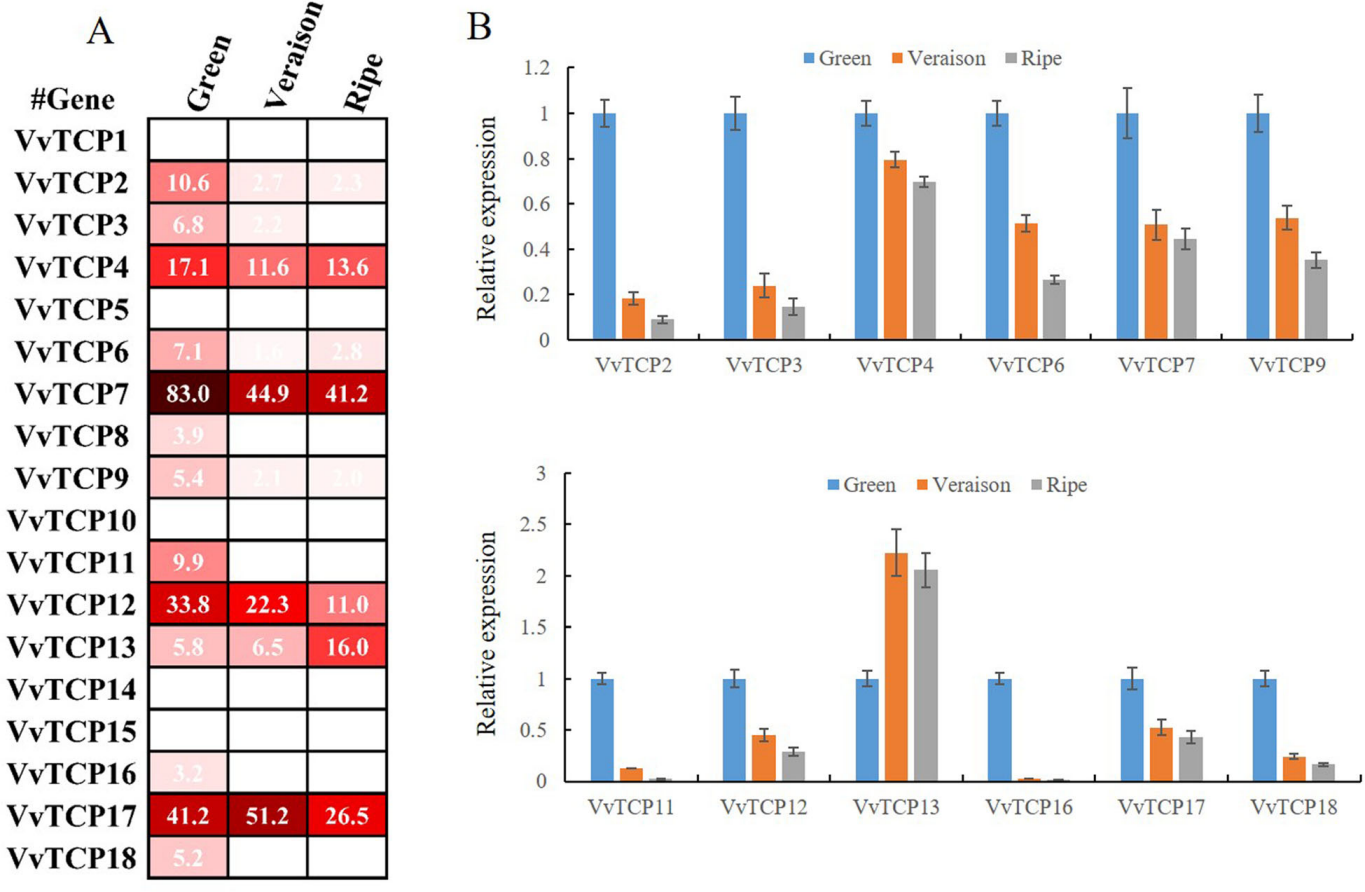
Expression profile of grapevine *VvTCP* genes during three fruit developmental stages. **a** Hierarchical clustering of the transcript accumulation profiles of 18 VvTCP genes during three berry developmental stages. **b** RT-qPCR transcript analysis of 12 selected VvTCP genes at three berry developmental stages. Berries from 3 year old ‘Fujiminori’ grapevine trees were sampled in triplicate at the fruit expanding (40DAF or DAF40), veraison (65DAF or DAF65), and ripe (90DAF or DAF90) stages throughout the growing season. The experiments were repeated three times and provided consistent results

To understand the potential function of *VvTCP* genes in berry development and ripening, the transcript accumulation patterns of 18 *VvTCP* genes were investigated during three fruit developmental stages in grapevine using the expression profiles from the GEO DataSets (GSE77218) [47]. As shown in Fig. 7a, five *VvTCP* genes (*VvTCP1*, *5*, *10*, *14* and *15*) were almost undetectable during the whole processes of berry development in grapevine (Fig. 7a, Additional file 6: Table S5). Eleven *VvTCP* genes (*VvTCP2*, *3*, *4*, *6*, *7*, *8*, *9*, *11*, *12*, *16* and *18*) displayed the highest expression levels at green fruit stage, and then showed decreasing trend from veraison till to ripe stage, indicating potential roles during early berry development. On the contrary, the expression of *VvTCP13* was increased gradually during three berry development stages.

In order to validate the expression pattern of *VvTCP* genes in the various developmental stages of the berry by microarray data, qRT-PCR analysis of 12 detectable *VvTCP* genes was further performed at three berry development stages. As was expected, qRT-PCR results were highly consistent with the RNA-Seq data except for *VvTCP17* (Fig. 7b). For example, *VvTCP2*, *VvTCP3* and *VvTCP7* showed a relatively high expression levels in green stage but decreased sharply in veraison stage, and then changed slightly from veraison to ripening stage (Fig. 7b). *VvTCP13* was significantly higher expression in at ripe stage than that in green stage. However, the expression profiles of *VvTCP17* did not correspond with RNA-Seq data. *VvTCP17* was relatively high expression in veraison berry from RNA-Seq data, whereas the qRT-PCR analysis showed the highest expression in green berry stage (Fig. 7b). All these results implied that *VvTCP* genes might be involved in grapevine fruit development.

**Fig. 8:**
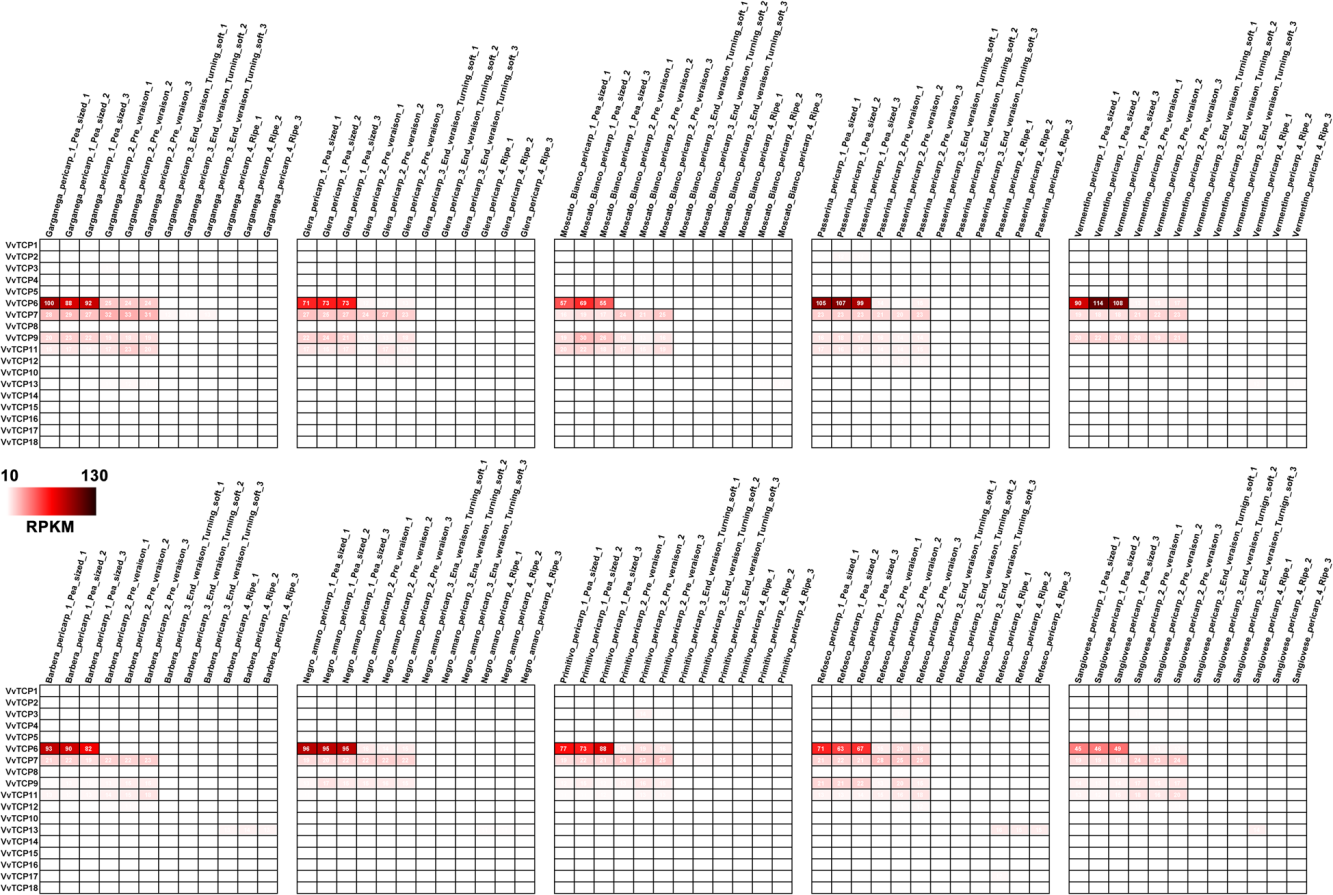
Expression profiles of the grapevine VvTCP genes in 10 different grapevine varieties at four berry developmental stages. Berries were sampled in triplicate at four developmental stages, the pea-sized berry stage at 20d after flowering, the berries beginning to touch stage just prior to veraison (Pre_veraison), the berry-softening stage at the end of veraison (End_veraison), and the fully ripe berry stage at harvest

To provide more information on the berry developmental and ripening functions of *VvTCP* genes in grapevine, we investigated their transcript accumulation patterns among 10 different grapevine varieties by using microarray data (accession numbers GSE62744 and GSE62745), which consists of four different fruit developmental stages (the pea-sized berry stage at 20d after flowering, the berries beginning to touch stage just prior to veraison, the berry-softening stage at the end of veraison, and the fully ripe berry stage at harvest [48]. As shown in Fig. 8, four detected *VvTCP* genes (*VvTCP6, 7*, 9 and *11*) were relatively higher expression in pea-sized berry and Pre_veraison stage and rapidly down-regulated during ripening, which were corresponded with the data from RNA-Seq and qRT-PCR analysis. Interestedly, *VvTCP6* were intensely expressed in pea-sized berry, implying that *VvTCP6* may play an important role during the early stages of grapevine berry development. Additionally, *VvTCP13* was only detected at ripe stage (Fig. 8), which indicated that *VvTCP13* might function in grapevine fruit ripening. All these results indicated that some *VvTCP* genes might play important roles in grapevine fruit development.

### Transcript profiling of *VvTCP* genes under various abiotic stress treatments

**Fig. 9:**
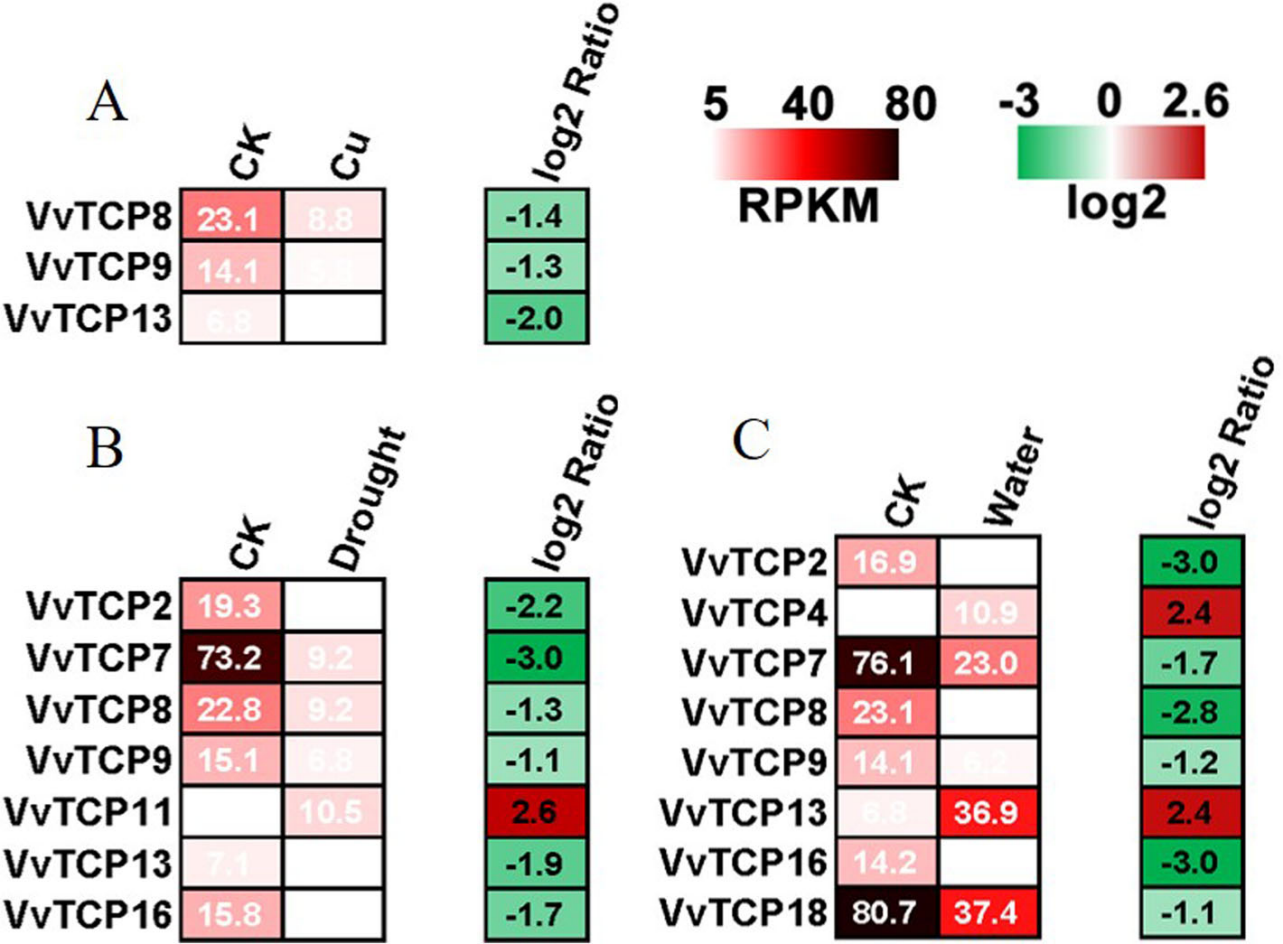
The expression of VvTCP genes under different abiotic stresses. **a**-**c**, Hierarchical cluster displaying the differentially expressed VvTCP genes under Cu, drought and waterlogging treatments. Data were obtained by RNA Sequencing and were expressed as Reads Per Kilobase of exon model per Million mapped reads (RPKM). The differentially expressed data were log2 transformed with R software. Blocks with green colors indicate decreased and red ones indicate increased transcription levels

Copper (Cu), salt, waterlogging and drought are common types of abiotic stresses in vineyards. To investigate the potential roles of the *VvTCP* genes in responses to different environmental stresses, the RNA-seq datas were collected for the 18 *VvTCP* genes in the leaves of the grapevine exposed to Cu, NaCl, waterlogging and drought treatment (Fig. 9, Additional file 7: Table S6). Overall, the *VvTCP* genes responded to waterlogging and drought stress to a greater extent than to Cu and NaCl treatment. For example, eight *VvTCP* genes were regulated in response to waterlogging treatment and seven *VvTCP* genes responded to drought stress (Fig. 9, Additional file 7: Table S6). In contrast, only three (*VvTCP8*, *9* and *13*) and one (*VvTCP3*) *VvTCP* genes were down-regulated expression in response to Cu and salinity stress, respectively, while the other *VvTCP* members were only slightly down-regulated or remained nearly unchanged (Fig. 9, Additional file 7: Table S6). Notably, three *VvTCP* genes (*VvTCP8*, *9* and *13*) responded to at least three treatments, indicating that these genes might be involved in multiple stress response processes. Moreover, the expression difference of *VvTCP* depended on the type of stress. *VvTCP13* was up-regulated in response to waterlogging stress, but was down-regulated in response to Cu and drought stress.

### Subcellular localization of VvTCP proteins

**Fig. 10:**
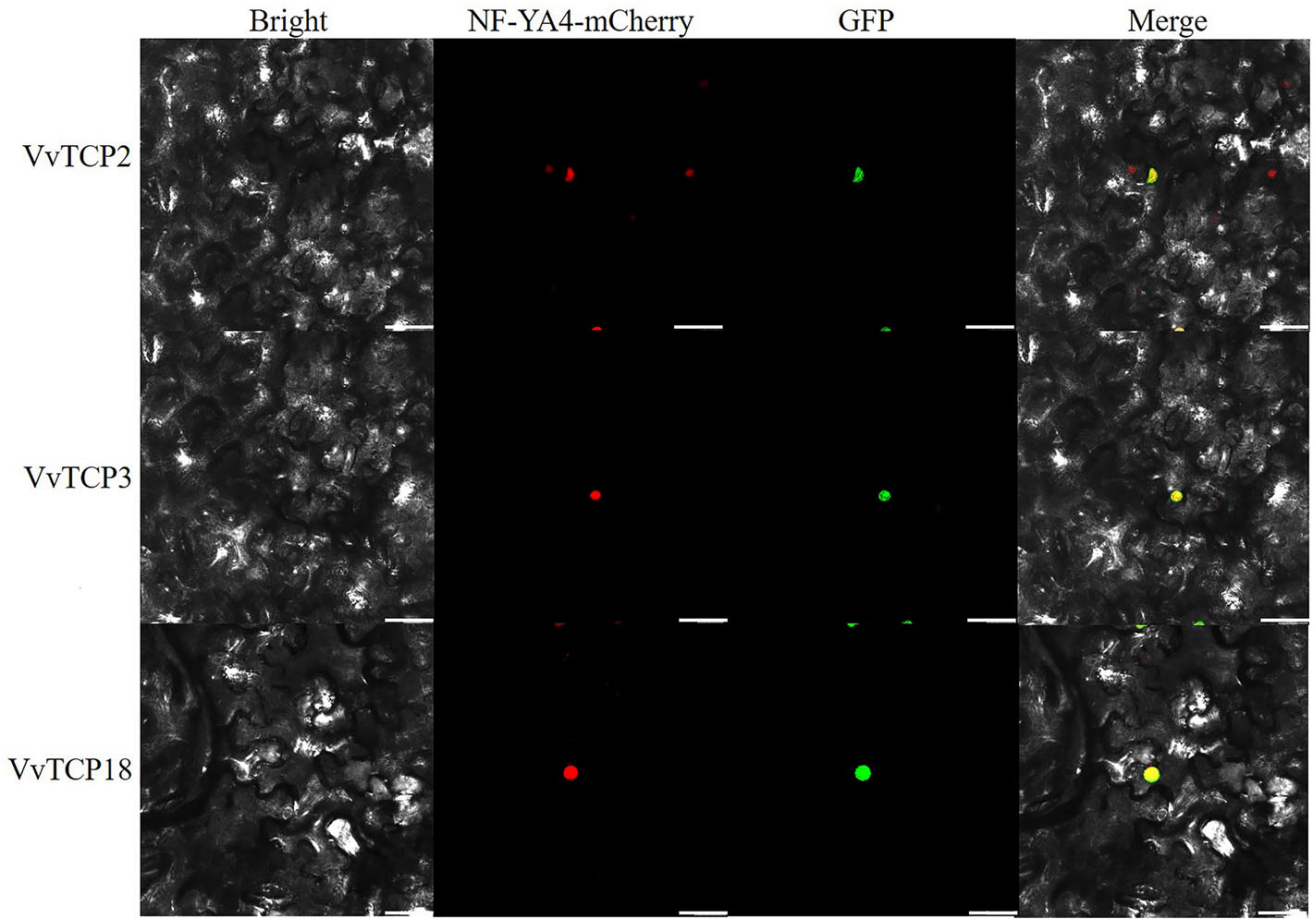
Subcellular localization of three GFP-fused grapevine TCP proteins. The three VvTCP-GFP fusion proteins (VvTCP2-GFP, VvTCP3-GFP, and VvTCP18-GFP) were transiently expressed in tobacco leaves and observed by confocal microscopy 72 h later. Nuclei were visualized by co-transformation of a mCherry-labelled nuclear marker (NF-YA4-mCherry). Scale bar, 50 μm

It is well know that the nuclear localization of transcription factors is very important for regulate the transcription of target genes by binding to specific cis-elements in their promoters. Previous studies have showed that TCP proteins were predominantly located in the nucleus, such as FvTCP8, FvTCP9 and FvTCP13 in strawberry. In this study, the majority of VvTCP proteins were predicted to be located on the nucleus by WoLF PSORT (Table 1). To characterize the subcellular localization of the VvTCP, three cloned VvTCP genes (VvTCP2-GFP, VvTCP3-GFP and VvTCP18-GFP) were introduced into the pCAMBIA1300 vector by CaMV 35S promoter. The recombinant three fusion constructs was infiltrated into *N. tabacum* epidermal cells. As indicated in Fig. 10, green fluorescence signals from the expressed fusion VvTCP2-GFP, VvTCP3-GFP and VvTCP18-GFP were specifically distributed within the nuclei as confirmed by a mCherry-labelled nuclear marker (NF-YA4-mCherry). These results showed that VvTCP2, VvTCP3 and VvTCP18 were nuclear proteins, and consistent with the prediction results and previous studies in strawberry [31].

## Discussion

The plant-specific TCP transcription factors are known to play important roles in diverse aspects of physiological and biological processes during plant growth and development. To date, the TCP gene family have been investigated and characterized in various plant species such as *Arabidopsis* [32], tomato [21], apple [33], strawberry [31] and peach [22]. However, virtually no systematic and comprehensive informations of the TCP gene family in grapevine, a nutritious and economically important fruit crop all over the world, have been undertaken. In present study, 18 non-redundant *VvTCP* genes were identified and analyzed from grapevine genome. Furthermore, we peformed a multi-level analysis of the *VvTCP* genes in grapevine by investigating their evolutionary relationships, gene structure, protein motifs, duplication events, cis-acting elements, expression profiles in different tissues and developmental stages and under various stress treatment. The systematic characterization of *VvTCP* genes in grapevine will provide a better foundation for further functional studies of this gene family during grapevine growth and development.

### Evolutionary conservation and divergence of the *VvTCP* gene family in grapevine

Phylogenetic analysis and sequence alignment showed that all 18 *VvTCP* were classified into three major subgroups, which was consistent with the previous described in *Arabidopsis*, rice, tomato and strawberry [21, 31, 54]. Each subgroups contained TCP genes from *Arabidopsis*, rice, tomato, strawberry and grapevine (Fig. 1a). Furthermore, *VvTCP* members from the same group or subgroup shared a similar motif composition and intron/exon organization. For example, motif 2 and 4 were only present in class I and class II subgroup, respectively (Fig. 3). The consistency of the motif compositions and the exon/intron structures of *VvTCP* genes further supported the close evolutionary relationships.

In addition, the number of TCP genes was relatively conserved among *Arabidopsis* (24 members), rice (22 members) and strawberry (19 members) (Fig. 1b). However, it was significantly smaller than that present in tomato (30 members) and apple (52 members), which was consistent with the genome sizes of tomato (960 Mb) [57] and apple (742 Mb) [58], implying that TCP genes in various plants have expanded to different degrees. It is found that many TCP genes in tomato and *Arabidopsis* had three counterparts in grapevine (Additional file 1: Figure S3 and Additional file 1: Figure S4), indicating that the expansion of TCP family in grapevine may be caused by genome duplication events such as segmental duplication and whole-genome duplication. Our analysis showed that the number of paralogous TCP gene pairs accounted for over 50% of the entire TCP gene family in grapevine (Fig. 4). The fact supported the view that segmental duplication was a predominant duplication event for TCP genes and the major contributor to the expansion of TCP gene family in grapevine, as described previously, in *Arabidopsi*s and cotton [59].

### Functional divergence of *VvTCP* genes in plant growth and development

Increasing evidences suggest that *TCP* gene family involved in a wide range of functions during plant growth and development processes such as in shoot branching, leaf development, flower development and senescence [2]. The expression pattern of *VvTCP* genes in 42 different grapevine organs/tissues were investigated using an expression atlas of *V.vinifera* cv Corvina [45]. The expression analysis indicated that some *VvTCP* genes can be classified together according to their expression abundant in specific tissues of grapevine, probably reflecting their involved in a common metabolic and/or developmental process.

Previous studies have shown that TCP genes play important roles in leaf senescence and development [60]. In *Arabidopsis*, five *CIN*-like subfamily of TCP (*AtTCP2*, *3*, *4*, *10* and *24*) were targeted by miRNA319 [61]. Ectopic expression of a miR319-insensitive *TCP4* (*mTCP4*) gene led to the formation of miniature leaves during early stages of leaf development [62]. Proper regulation by miR319a of *TCP4* was also important for petal and stamen development [14, 63]. Furthermor, overexpression of miR319 or inhibition of multiple *CIN*-like TCP genes leat to delayed senescence, whereas overexpression of *CIN*-like TCP genes accelerates leaf senescence by activating biosynthesis of the hormone jasmonic acid [26, 64]. In grapevine, *VvTCP5*, *VvTCP6* and *VvTCP18*, the three closest homologs of these *Arabidopsis CIN*-like genes, had putative binding sites for VvmiR319b (Fig. 2c). *VvTCP5* was only relatively high expression level in senescing leaf (Fig. 6), implying its potential function in leaf senescence. *VvTCP6* and *VvTCP18* were expressed at high levels in young leaf, mature leaf, young inflorescence, bud after burst and burst bud (Fig. 6), which were in agreement with the previous reports that *CIN*-like TCP genes and its post-transcriptional regulator miR319 play pivotal roles in leaf and flower development. These observations suggested that these miRNA-targeted *VvTCP* genes were likely to perform similar roles in leaf and flower development in grapevine to those of the Arabidopsis homologs.

The CYC/TB1 subgroup is mainly participated in the axillary meristems development [2]. In *Arabidopsis*, *AtTCP1*, the most closely homolog of CYC, was involved in the longitudinal elongation of leaves. *AtTCP1* was strong expression in the petiole, lower portion of the inflorescence stem, and the midrib and distal region of expanding rosette leaves [65]. *VvTCP10*, which was closely homology with *AtTCP1*, was transcribed at relatively high levels in young inflorescence, green stem, bud after burst, burst bud or well-developed inflorescence and was almost undetectable in other tested tissues (Fig. 6). This result was partly consistent with the expression profile of *AtTCP1* in *Arabidopsis*, indicating that *VvTCP10* might play roles in flower and bud development in grapevine. *AtTCP18* acted downstream of auxin and strigolactone to coordinate axillary bud outgrowth and up-regulation of *AtTCP18* led to an inhibition of lateral branching. In contrast, mutation of *AtTCP18* resulted in an increased number of rosette branches. *AtTCP12* displayed a weaker or no mutant phenotype compared with *AtTCP18* [9, 66]. The *VvTCP1*, the homolog of the *AtTCP12*, was almost undetectable in all tissues, which implied that *VvTCP1* was a potential functional redundant TCP member.

By contrast, most Class I genes, which usually play roles in cell growth and proliferation, exhibited more widespread and less tissue-specific expression patterns, such as in leaf, flower, stem and fruit (Fig. 6). These findings suggested that these Class I *VvTCP* genes might play various regulatory roles at multiple growth and development process. For example, *AtTCP14* and *AtTCP15* were involved in cell proliferation during seed, leaf and internode development [12, 16]. *AtTCP16* was proposed to modulate early pollen development [20]. *AtTCP19* and *AtTCP20* negatively regulated the onset of leaf senescence by jasmonate signaling pathway [7, 17]. All of these *AtTCP* genes had at least one counterpart in grapevine, indicating that Class I TCP in grapevine might share similar functions with *Arabidopsis* homologs. Taken together, the above-mentioned results from model plants underlined that the TCP family members performed diverse biological functions in multiple plant growth and development processes.

### Potential roles of *VvTCP* genes during berry development and ripening

Fruit development and ripening is a complex process and requires highly coordinated developmental events which were mainly controlling by a set of TFs regulatory networks [67]. In grapevine, all three genes from CYC/TB1 subgroup, *VvTCP1*, *VvTCP10*, and *VvTCP15* were not expressed in grapevine fruit (Fig. 7a), implying that these three genes in CYC/TB1 subgroup were rarely related to berry development and ripening. Similarly, some *SlTCP* genes in CYC/TB1 subgroup, such as *SlTCP7*, *SlTCP8*, *SlTCP9* and *SlTCP22*, were almost undetectable in fruits [21], indicating that TCP genes in CYC/TB1 subgroup might not be associated with fruit development and ripening in tomato and grapevine. Interesting, the CYC/TB1 subgroup genes in strawberry, *FvTCP6* and *FvTCP14*, showed the increased expression during the berry ripening process, and overexpression *FvTCP9* transiently by agro-infiltration in strawberry fruits effectively increased the expression levels of ripening-related genes [31], which suggested that they might be involved in strawberry fruit ripening. Taken together, the *TCP* genes in CYC/TB1 might play variable roles in fruits development and ripening of different species.

Additionally, little was known about the functions of CIN clade members in fruit development. For example, *PpTCP.C1* and *PpTCP.D1.1* were high transcript accumulation levels in early fruits in peach, but were not associated with fruit ripening, suggesting that these two CIN clade members were likely to be involved in early peach fruit development. In this study, the CIN clade included five *TCP* members in grapevine, and of these genes, *VvTCP5* was not expressed in grapevine fruits, indicating that *VvTCP5* was irrelevant to grapevine fruit development and ripening. Moreover, four *VvTCP* genes in CIN clade was also relatively high expression in early grapevine fruit (Fig. 7), which were generally in agreement with the expression profile of *PpTCP.C1* and *PpTCP.D1.1* in peach. The similar expression patterns suggested that *VvTCP* genes in CIN clade was likely to perform roles similar in early fruit development in grapevine.

In tomato, three *TCP* genes in PCF (class I) subgroup, including *SlTCP12*, *SlTCP15*, and *SlTCP18*, were dominantly expressed in tomato fruits, implying that these *TCPs* were likely to play important roles during fruit development and ripening [21]. In present study, nine out of ten *VvTCP* genes in PCF subgroup were down-regulated expression during the berry ripening process, except for *VvTCP13* (Fig. 7), indicating that these *VvTCP* genes might play a regulatory role during the early stages of berry development. Of these nine genes, *VvTCP7*, *VvTCP9* and *VvTCP12* which were individually homologous of *SlTCP15*, *SlTCP12* and *SlTCP18*, might be involved in fruit development, due to the high levels of expression in the developing grapevine fruit (Figs. 7 and 8). In peach, the expression of *PpTCP.A2* was negatively correlated to fruit ripening, and silencing of *PpTCP.A2* enhanced the expression of *PpACS1* and increased ethylene production, indicating that *PpTCP.A2* was probably involved in fruit ripening by regulating ethylene biosynthesis [22]. Similarly, the expression of *VvTCP2*, which was a homologous gene of *PpTCP.A2*, was consistent with the expression profile of *PpTCP.A2* and implied that the *VvTCP2* gene was likely to play similar roles to *PpTCP.A2* in grapevine fruit development and ripening.

## Conclusions

In conclusion, 18 *VvTCP* genes were identified in the grapevine genome, which were distributed on 11 chromosomes. These *VvTCP* genes were divided into two classes based on the phylogenetic and structural feature. A lot of cis-acting elements were observed in the *VvTCP* promoter sequences, implying that *VvTCP* gene were controlled by a complex regulatory network. *VvTCP* genes might play important roles during grapevine growth and development as indicated by their spatial and temporal expression patterns. Notably, most *VvTCP* genes in grapevine were higher expressed in fruitlets than in other developmental and ripening fruits, indicating that these *VvTCP* genes were probably involved in early development in grapevine fruit. Taken together, all these findings will lay a solid foundation for for further unraveling the functions of *VvTCP* genes in grapevine growth and development.

## Supplementary information

**Additional file 1: Figure S1.** Chromosomal distribution of *VvTCP* genes. Chromosome numbers are provided at the top of each chromosome together with the approximate size. **Figure S2.** The conserved protein motifs in the VvTCP proteins. The x-axis indicates the conserved sequences of the domain. The height of each letter indicates the conservation of each residue across all proteins. The y-axis is a scale of the relative entropy, which reflects the conservation rate of each amino acid. **Figure S3.** Synteny analysis of TCP genes between *Arabidopsis* and grapevine. The chromosomes of grapevine and *Arabidopsis* are depicted as a circle. The approximate distribution of each AtTCP gene and VvTCP gene is marked with a short line on the circle. Red curves denote the details of syntenic regions between grapevine and Arabidopsis TCP genes. **Figure S4.** Synteny analysis of grapevine and tomato *TCP* genes. The chromosomes of grape and tomato are depicted as a circle. The approximate distribution of each *VvTCP* gene and *SlTCP* gene is marked with a short line on the circle. Red curves denote the details of syntenic regions between grapevine and tomato TCP genes.

**Additional file 2: Table S1.** The primers sequences of VvTCP genes for qRT-PCR and gene cloning.

**Additional file 3: Table S2.** The synteny regions amony grapevine, Arabidopsis and tomato TCP genes.

**Additional file 4: Table S3.** Promoter analysis of the grapevineTCP gene family.

**Additional file 5: Table S4.** Tissue-specific expression patterns of VvTCP genes in grapevine.

**Additional file 6: Table S5.** Expression profiles of the grapevine VvTCP genes during three fruit developmental stages.

**Additional file 7: Table S6.** Expression profiles of the grapevine VvTCP genes in response to abiotic stress.

## Data Availability

Microarray data is available as GEO accession number GSE36128 (http://www.ncbi.nlm.nih.gov/geo/). RNA-Seq data is available as GEO accession number GSE77218 (https://www.ncbi.nlm.nih.gov/geo/query/acc.cgi), GSE62744 (https://www.ncbi.nlm.nih.gov/geo/query/acc.cgi) and GSE62745 (https://www.ncbi.nlm.nih.gov/geo/query/acc.cgi).

## Abbreviations

bHLH: Basic helix-loop-helix
CNR: Colorless non-ripening
CYC: Cycloidea
GEO: Gene expression omnibus
GFP: Green fluorescent protein
GRAVY: Grand average of hydropathicity
MW: Molecular weight
PCF: Proliferating cell nuclear antigen factor
pI: Isoelectric points
RIN: Ripening inhibitor
RNA-seq: RNA sequencing
RPKM: Reads per kilobase per million mapped reads
TAIR: *Arabidopsis* information resource
TB1: Teosinte branched1
