# Supplementary material for: Genome-wide identification and transcript analysis of TCP transcription factors in grapevine
Source: BMC Genomics. 2019 Oct 29;20:786. doi: 10.1186/s12864-019-6159-2 (PMC6819353; doi:10.1186/s12864-019-6159-2)
Supplement: Supplementary file 1 — Additional file 1: Figure S1. Chromosomal distribution of VvTCP genes. Chromosome numbers are provided at the top of each chromosome together with the approximate size. Figure S2. The conserved protein motifs in the VvTCP proteins. The x-axis indicates the conserved sequences of the domain. The height of each letter indicates the conservation of each residue across all proteins. The y-axis is a scale of the relative entropy, which reflects the conservation rate of each amino acid. Figure S3. Synteny analysis of TCP genes between Arabidopsis and grapevine. The chromosomes of grapevine and Arabidopsis are depicted as a circle. The approximate distribution of each AtTCP gene and VvTCP gene is marked with a short line on the circle. Red curves denote the details of syntenic regions between grapevine and Arabidopsis TCP genes. Figure S4. Synteny analysis of grapevine and tomato TCP genes. The chromosomes of grape and tomato are depicted as a circle. The approximate distribution of each VvTCP gene and SlTCP gene is marked with a short line on the circle. Red curves denote the details of syntenic regions between grapevine and tomato TCP genes. [file 12864_2019_6159_MOESM1_ESM.docx]

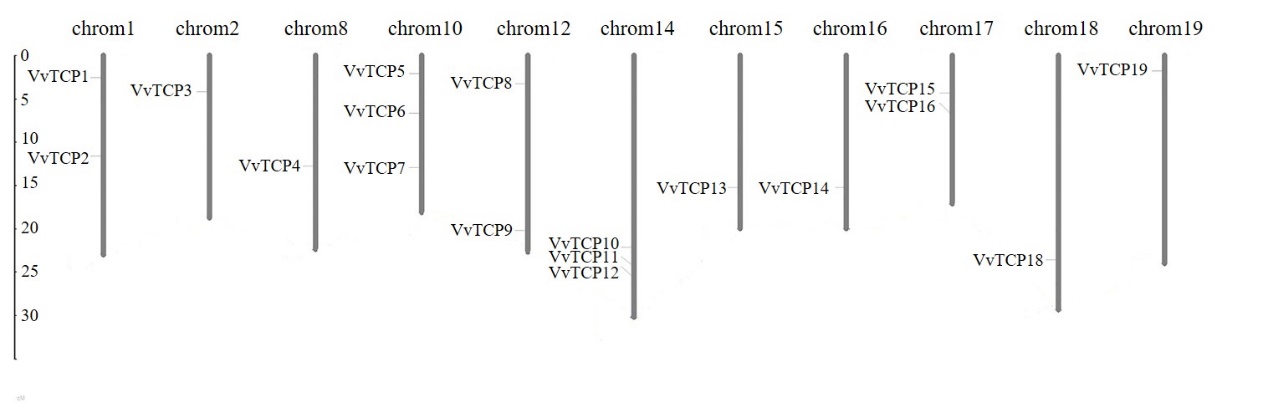


Figure S1. Chromosomal distribution of *VvTCP* genes. Chromosome numbers are provided at the top of each chromosome together with the approximate size.


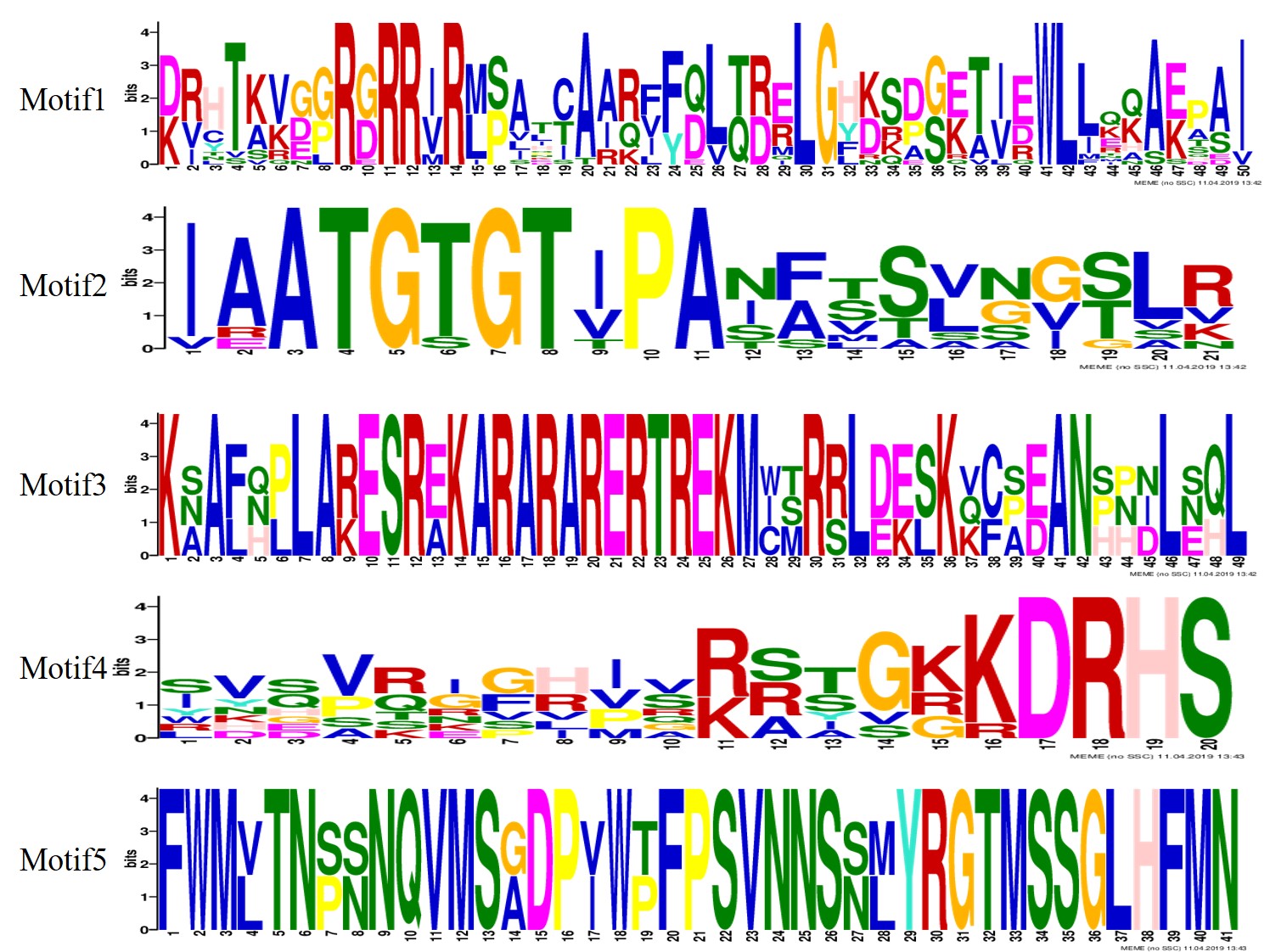


Figure S2 The conserved protein motifs in the VvTCP proteins. The x-axis indicates the conserved sequences of the domain. The height of each letter indicates the conservation of each residue across all proteins. The y-axis is a scale of the relative entropy, which reflects the conservation rate of each amino acid.


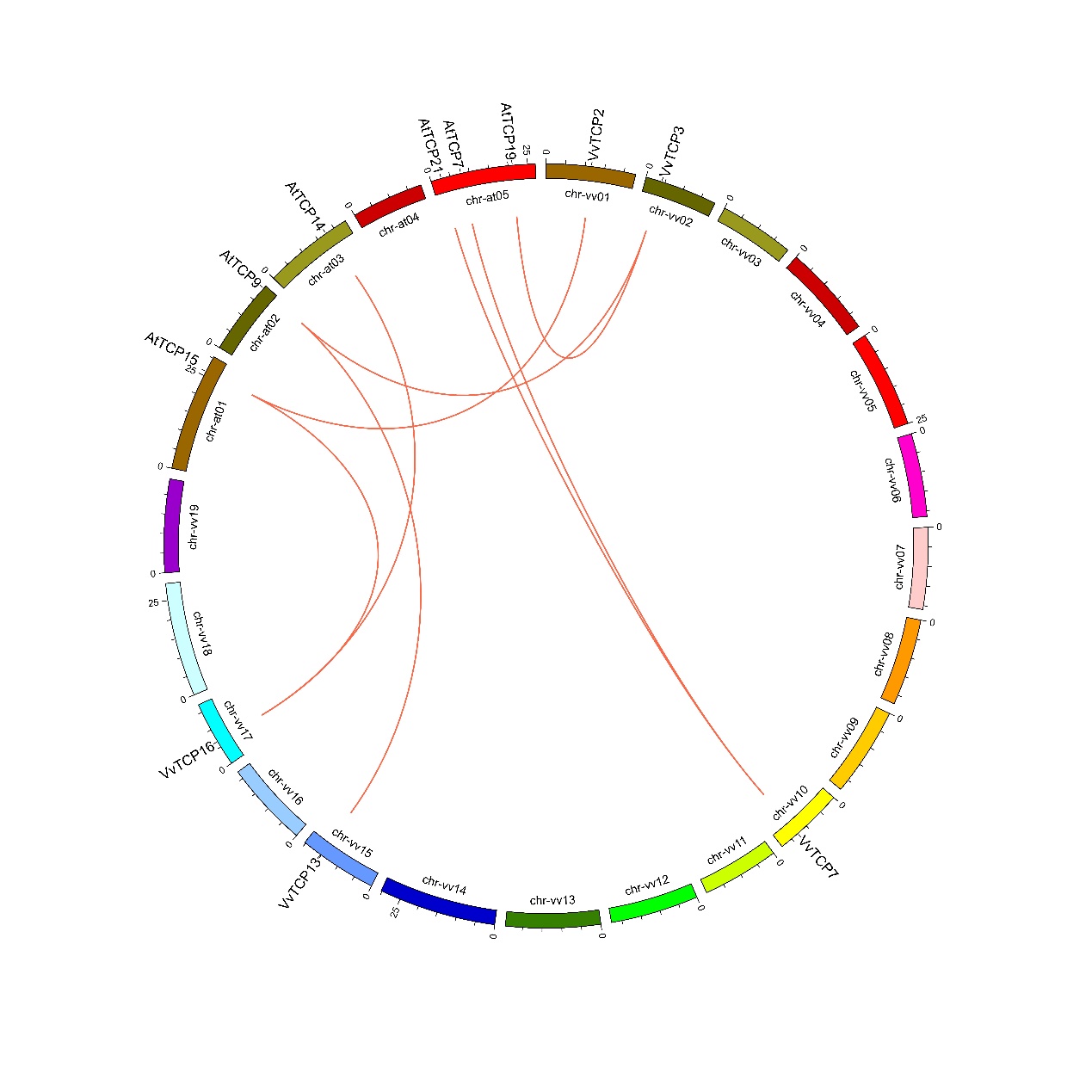


Figure S3 Synteny analysis of TCP genes between Arabidopsis and grapevine. The chromosomes of grapevine and *Arabidopsis* are depicted as a circle. The approximate distribution of each AtTCP gene and VvTCP gene is marked with a short line on the circle. Red curves denote the details of syntenic regions between grapevine and Arabidopsis TCP genes.


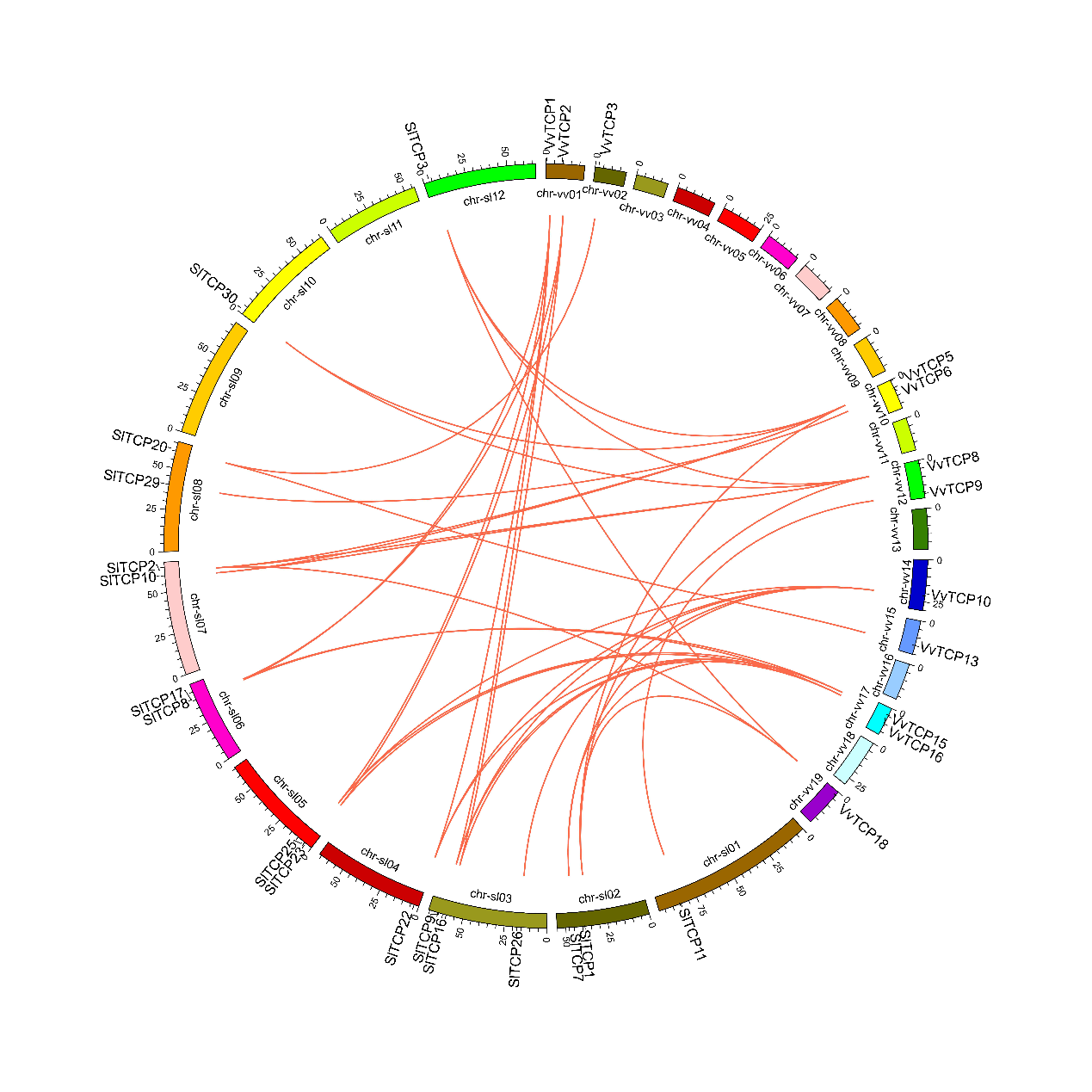


Figure S4 Synteny analysis of grapevine and tomato *TCP* genes. The chromosomes of grape and tomato are depicted as a circle. The approximate distribution of each *VvTCP* gene and *SlTCP* gene is marked with a short line on the circle. Red curves denote the details of syntenic regions between grapevine and tomato TCP genes.
